# Isolation, Identification and Antimicrobial Resistance Analysis of Canine Oral and Intestinal *Escherichia coli* Resistant to Colistin

**DOI:** 10.3390/ijms241713428

**Published:** 2023-08-30

**Authors:** Hui-Hua Zheng, Chao Yu, Xin-Yue Tang, Chong-Tao Du, Guang-Hong Xie

**Affiliations:** 1 College of Veterinary Medicine, Jilin University, Changchun 130062, China; 2 College of Animal Science and Technology and College of Veterinary Medicine, Zhejiang A & F University, 666 Wusu Street, Lin’an District, Hangzhou 311300, China

**Keywords:** dog, colistin resistance, *Escherichia coli*, antibiotic susceptibility testing

## Abstract

In recent years, the antimicrobial resistance in *Escherichia coli* has gradually developed into a global problem. These resistant bacteria could be transmitted to humans through animal feces in the environment or direct contact with pets, leading to a problem in bacterial treatment for humans and animals. Now, the antibiotic resistance of oral and intestinal microbiota from dog origins remains unclear in China. Therefore, this study first analyzed the current colistin resistance of oral and intestinal microbiota from dog origins in mainland China. A total of 536 samples were collected from dogs in mainland China and, respectively, cultured on the SS and MacConkey agar plate containing colistin (4 μg/mL) to obtain bacteria, and the antibiotic-resistance phenotype of *Escherichia coli* was investigated for nine antibiotics. Results showed that a total of *2259* colistin-resistant bacteria were isolated from samples and identified, and among them, the isolated rate of *Escherichia coli* (34.01%, 769/2259) was relatively higher than that of other bacteria. Subsequently, it was found that the resistance of these *Escherichia coli* was very severe by exploring its resistance to different antibiotics, particularly to three common antibiotics in a clinic which were ceftriaxone, ampicillin and trimethoprim/sulfamethoxazole, with the resistance rates of 60.60% (466/769), 57.22% (440/769), and 53.06% (408/769), respectively. Moreover, the simultaneous resistance of *Escherichia coli* to one or more antibiotics was determined, and 69.96% (538/769) strains have defined the resistance to both two or more antibiotics, and even 13 of *Escherichia coli* strains that were resistant to all nine antibiotics, indicating that the *Escherichia coli* from dog origins has severe antibiotic resistance in the clinic. In conclusion, this study guided the use of antibiotics and could draw attention to antibiotic resistance in veterinary clinical treatment for animals in the future.

## 1. Introduction

Antimicrobial agents are used to treat infectious diseases in both humans and animals and in many instances, the same drug or drug classes are used. Antimicrobial resistance may follow antimicrobial use. Several lines of evidence link antimicrobial-resistant human pathogens to foodborne pathogens of animal origin, including direct epidemiologic studies, temporal evidence, additional circumstantial evidence, trends in antimicrobial resistance among Salmonella isolates and trends in antimicrobial resistance among other pathogens [1]. Extended-spectrum β-lactamase (ESBL)-producing Gram-negative bacilli have been reported from companion animals, and Ljungquist et al. reported on the household transfer of ESBL/ampC-producing *Enterobacteriaceae* between humans and dogs [2,3]. Close contact between cats, dogs, and their owners may lead to the occurrence and transmission of antibiotic-resistant microorganisms [4].

Colistin, also known as polymyxin E, belongs to a cyclic polypeptide antibiotic produced by *Bacillus polymyxa*, which was limited use owing to its renal and neurotoxicity in the first introduction [5]. Renewed interest in this drug related to its activity against multidrug-resistant Gram-negative bacilli, and therefore, its clinical utility. In veterinary medicine, it can be used in pets for the prevention and treatment of bacterial infections [6]. Because colistin has been called a last-resort antibiotic, its resistance concerns are escalating. In particular, transferable polymyxin resistance reported from animals, food and humans in China in 2015 solidified the magnitude of these concerns [7]. It was confirmed that the overuse of colistin has been shown to cause colistin resistance in bacteria colonizing the intestinal gut of animals [6]. Colistin reuse has resurfaced due to necessity and even more limited treatment options, particularly since the spread of multidrug-resistant bacteria (MDR) and carbapenem-resistant bacteria [8]. Colistin has been used in veterinary medicine for decades as a common therapy in livestock, but the data regarding colistin resistance are relatively scarce [7]. In a previous study, the colistin resistance gene mcr-1-producing bacteria have been reported in zoonotic transmission from pets to humans who adopt them for protection, entertainment, or companionship [9]. In recent years, with people’s higher pursuit of spiritual level, companion pet dogs that can provide good emotional value for humans have been raised by more and more families in China, and pets are in daily close contact with humans [10]. Currently, colistin-resistant bacteria have become a significant public health issue. Therefore, determining the prevalence of colistin-resistant bacteria in pets is critical to identifying any potential risk factors for colistin-resistance transmission, particularly zoonotic transmission of bacteria [11].

Nevertheless, up to now, colistin-resistant strains in the oral and fecal origin of pet dogs have never been investigated in China, according to the literature. Therefore, in this study, we aimed to investigate and analyze the situation of drug-resistant bacteria carried in the oral and intestinal cavities of dogs for a better understanding of the colistin-resistant bacteria found in pet dogs.

## 2. Results

### 2.1. Sample Information

In this study, a total of 536 fecal or oral samples were collected from dogs which contained a female ratio of 55.41% (297/536) on the gender. In terms of the dog breeds, twenty-three breeds were used to collect their fecal or oral samples, including poodle (30.22%, 162/536), bichon fries (12.87%, 69/536), pomeranian (7.84%, 42/536), schnauzer (6.53%, 35/536), chinese rural dog (5.60%, 30/536), Labradors (4.85%, 24/536), golden retriever (4.48%, 24/536), welsh corgi pembroke (3.54%, 19/536), border collie (3.17%, 17/536), shiba inu (2.43%, 13/536), french bulldog (2.24%, 12/536), Samoyed (2.24%, 12/536), siberian husky (2.05%, 11/536), alaskan malamute (1.87%, 10/536), pug (1.31%, 7/536), papillon (1.12%, 6/536), beagle (1.12%, 6/536), chihuahua (0.93%, 5/536), yorkshire terrier (0.93%, 5/536), greyhound (0.75%, 4/536), cocker (0.37%, 2/536), and others (5.34%, 19/536). Taking their body condition, there were 83.02% (445/536) of healthy dogs and 16.98% (91/536) of diseased dogs and based on these diseases, some dogs (12.31%, 66/536) have been treated with antibiotics within two months which mainly included cephalosporins, tamoxifen, penicillin, ampicillin, and kanamycin. Additionally, the collection quantity in different regions displayed diversity, and their rates of 43.66% (234/536), 24.44% (131/536), 18.47% (99/536) and 13.43% (72/536) were observed in the Northern China, Central China, Western China and Eastern China, respectively. Moreover, these samples were collected from dogs of different age groups, and divided into three phases, such as the infancy (50.75%, 272/536), middle age (36.38%, 195/536), and senile period (12.87, 69/536). 

### 2.2. Screening for Colistin-Resistant Bacteria

After the incubation with the fecal samples, a total of 1456 colistin-resistant bacteria were obtained in this study. The results of PCR identification showed that expected PCR products were amplified from these colistin-resistant bacteria with the universal primers 27F and 1429R. On the basis of sequencing results, five different bacterial species were identified, including *Escherichia coli*, Klebsiella pneumoniae, Enterobacter cloacae, Salmonella and Proteus mirabilis (Table 1), and among them, *Escherichia coli* was the most common bacterial strains, with a ratio of 40.73% (593/1456). Additionally, after inoculating the enriched oral swabs into the SS and MacConkey agar plate containing colistin (4 μg/mL) for 24 h, a total of 803 drug-resistant strains were isolated and purified. Subsequently, three bacterial types were determined by PCR amplification and 16S rDNA sequencing. The isolate rates from high to low were Proteus mirabilis (67.37%, 541/803), *Escherichia coli* (21.92%, 176/803) and Klebsiella pneumoniae (10.71%, 86/803) (Table 1).

### 2.3. Isolation of Colistin-Resistant Escherichia coli from Dog Origin

According to statistical analysis, the isolated rate of colistin-resistant *Escherichia coli* (34.01%, 769/2259) was relatively higher than that of other bacteria of dog origin. The colistin-resistant *Escherichia coli* from dog origin were analyzed based on different factors, such as the sampling origin, living area and the use of antibiotics in dogs (Table 2). Among these strains, 593 colistin-resistant *Escherichia coli* were isolated from intestinal samples and 176 strains from oral swabs. Based on the analysis of antibiotic usage in dogs, 86.74% (667/769) of those colistin-resistant *Escherichia coli* strains were isolated from dogs that did not use antibiotics within two months. In addition, the isolate rates of colistin-resistant *Escherichia coli* were 43.43% (334/769), 22.37% (172/769), 21.07% (162/769) and 13.13 (101/769) in the Northern China, Central China, Western China and Eastern China, separately.

### 2.4. Drug Sensitivity Test

By exploring the resistance of *Escherichia coli* to different antibiotics, it was found that the resistance of these *Escherichia coli* was very severe (Table 3). These *Escherichia coli* from dog origin are highly resistant to three common antibiotics in the clinic, which were ceftriaxone, ampicillin and trimethoprim/sulfamethoxazole, and their drug resistance rates were up to 60.60% (466/769), 57.22% (440/769), and 53.06% (408/769), respectively. Although the drug resistance to myxin, imipenem and meropenem was lower than these of the three common antibiotics, their drug resistance rate could also reach 26.92% (207/769), 15.86% (122/769), 11.31% (87/769), individually. 

**Figure 1:**
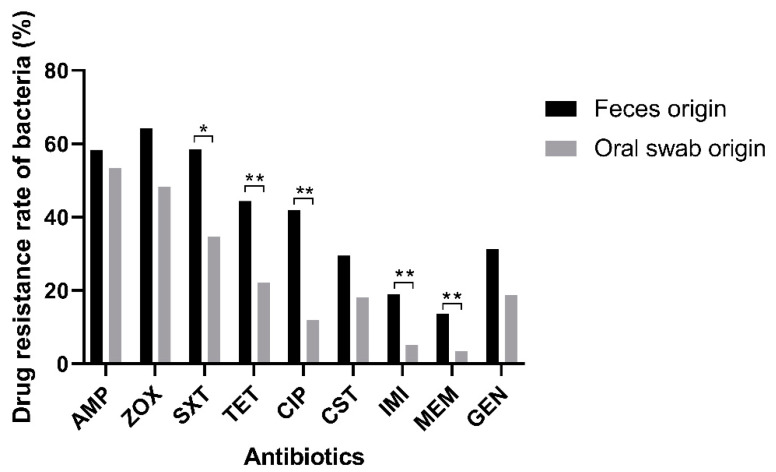
Resistance of *Escherichia coli* isolated from the oral and intestinal samples of dog origins to different antibiotics. Statistically significant differences (*p* < 0.05) are indicated by *, and statistically extremely significant differences (*p* < 0.01) are indicated by **.

Subsequently, the drug resistance differences of *Escherichia coli* from oral and fecal samples of dogs were analyzed in this study (Figure 1). It was seen that the trend of antibiotic resistance rate of *Escherichia coli* isolated from fecal samples was relatively consistent with that from the oral swabs and displayed a high resistance rate to common clinical antibiotics and a lower rate to newly applied antibiotics. However, the antibiotic resistance rate of *Escherichia coli* isolated from fecal samples was significantly higher than that from the oral swabs, especially to Trimethoprim/sulfamethoxazole, tetracycline, Ciprofloxacin, Imipenem, and Meropenem (*p* < 0.05 or *p* < 0.01).

**Figure 2:**
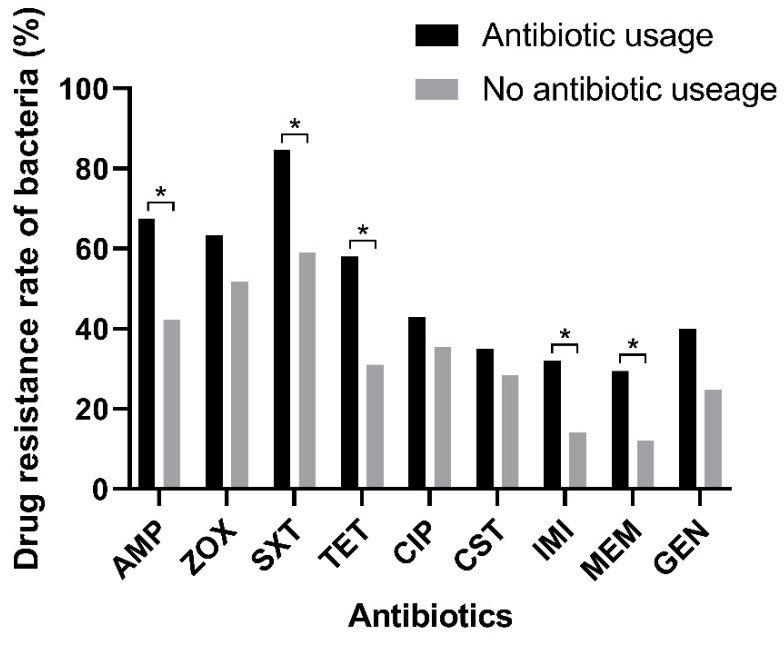
Resistance of *Escherichia coli* from dog origins to different antibiotics based on the antibiotic usage histories of dogs. Statistically significant differences (*p* < 0.05) are indicated by *.

According to the drug resistance analysis results of *Escherichia coli* isolated from dogs with antibiotic usage histories to different drugs, it was confirmed that the use of antibiotics had a significant impact on the resistance of *Escherichia coli* (Table 4). Among these *Escherichia coli* strains isolated from dogs that have used antibiotics in the past two months, the resistance rates of nine drugs were all higher than those of the strains isolated from dogs without antibiotics. The drug resistance rates of ampicillin, Trimethoprim/sulfamethoxazole, tetracycline, Imipenem and Meropenem were statistically different between the two groups (*p* < 0.05 or *p* < 0.01) (Figure 2).

**Figure 3:**
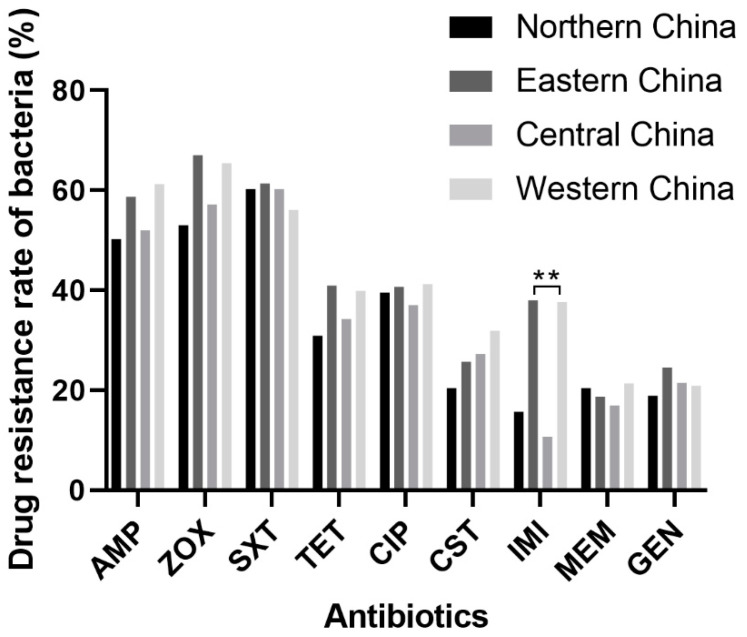
The antibiotic resistances of *Escherichia coli* isolated from dogs of different regions. Statistically extremely significant differences (*p* < 0.01) are indicated by **.

Moreover, the drug resistance of *Escherichia coli* isolated from samples which were collected from different regions was analyzed in this experiment (Table 4). The results showed that the impact of different sampling regions on the drug resistance of *Escherichia coli* was not significant, and the trend of resistance to several antibiotics in this survey was similar in each sampling region. Notably, the drug resistance of *Escherichia coli* in Eastern China was statistically different from that in Western China (*p* < 0.05) (Figure 3). 

### 2.5. Multidrug Resistance Statistics of Escherichia coli

Based on the statistical data in this study, the results of the simultaneous resistance of *Escherichia coli* from dog origins to one or more antibiotics are shown in Table 5. It was seen that there were 40 types of antimicrobial spectrum of *Escherichia coli* from dog origins. Among the 769 strains of *Escherichia coli* obtained in this study, only 46 bacterial strains were sensitive to nine antibiotics, accounting for 5.98% (46/769). It was the most common resistance to both antibiotics simultaneously, and of 216 strains were defined the resistance to both antibiotics, with a ratio of 28.09% (216/769). Surprisingly, the proportion of these *Escherichia coli* strains with multiple drug resistance (antibiotic resistant types ≥ 3) reached 41.87% (322/769), and namely, more than 40% of *Escherichia coli* from dog origins were multidrug-resistant strains currently. Furthermore, there were 11.96% (92/769) of *Escherichia coli* strains were resistant to six or more types of antibiotics, and we found even 13 *Escherichia coli* strains that were resistant to all nine antibiotics, indicating that the *Escherichia coli* from dog origins have severe antibiotic resistance in the clinic.

## 3. Discussion

Multiple antibiotic-resistant bacteria have become a public health threat to humans and animals, and their prevalence in humans and animals is often related to persistent infection, increased incidence rate and mortality of complications [12]. In a previous study, it has been observed that there may be differences in the frequency of antibiotic resistance when comparing fecal microbiota from different origins in dogs [13]. In recent years, antibiotic-resistant and pathogenic bacteria from dog origins have received attention. In this study, the fecal and oral samples were from dogs of different breeds, which mainly were small dogs in somatotype, particularly the poodle. Interestingly, we also found a higher proportion of small dogs than medium or large breeds in other research [14,15,16,17], and it might be a reason that the small size category was the most frequent breed raised by humans owing to its convenience for feeding and cute appearance. Besides, these fecal and oral samples were taken from dogs that were of different age phases and lived in different regions covering the entire mainland China, avoiding the differences in results caused by regions or ages. Usually, dogs should have used no antibiotic therapy in a screening of antibiotic-resistant bacteria [18], which might be due to the use of antibiotics leading to false positives. Hence, the samples were mainly collected from dogs without antibiotic therapy within two months in this study. Although the types of antibiotics used in dogs were similar to those in humans, the use of antibiotics might even be more extensive and widespread at veterinarian clinical, and the common antibiotics included β lactams, macrolide, aminoglycosides and tetracyclines [19,20]. Several studies indicated that members of the oral microbiota were involved in intestinal dysbiosis, indirectly affecting the composition of the intestinal microbiota via dissemination into the gut [21,22,23]. It was demonstrated that the majority (54%) of the patient-enriched, taxonomically assigned members of intestinal microbiota originated from the oral cavity by using metagenomics and gene catalogs [24]. As two common bacterial communities, we collected fecal samples and oral swabs of dogs to analyze their antibiotic resistance in this study. A total of 2259 colistin-resistant bacteria were isolated from samples and identified, and among them, the isolated rate of *Escherichia coli* (34.01%, 769/2259) was relatively higher than that of other bacteria, which was consistent with the result that the detection rate of *Escherichia coli* of cloacal swabs from animal origin was the highest in the previous study [25]. Other bacteria were detected in fecal samples and oral swabs other than *Escherichia coli*, including *Proteus mirabilis*, *Klebsiella pneumoniae*, *Enterobacter cloacae* and *Salmonella*, and these isolated bacterial species all belonged to the phylum of Proteobacteria, the class of γ-proteobacteria, the order of Enterobacteriales, the family of *Enterobacteriaceae*. In a previous study, Omatsu et al. showed that four phyla Firmicutes, Proteobacteria, Bacteroidetes, and Fusobacteria were dominant in the intestinal microbiota of small dogs, and in the family level of microbiota in poodles, Enterobacteriaceae in Proteobacteria, Bacteroidaceae in Bacteroidetes, and Lachnospiraceae in Firmicutes were predominant in healthy poodles [26]. Moreover, Pilla and Suchodolski displayed that most bacterial sequences identified in the canine gastrointestinal tract fall into five phyla: Firmicutes, Proteobacteria, Fusobacteria, Bacteroidetes, and Actinobacteria [27]. From this, it was seen that the extensive colistin resistance of the family *Enterobacteriaceae* has brought a serious threat to the drug treatment of dogs in veterinary clinics. Research has shown that the drug-resistant Gram-negative bacteria *Escherichia coli*, *Proteus mirabilis*, and *Klebsiella pneumoniae* belong to the common conditioned pathogens clinically and could cause a wide range of infections [28]. With the abuse of clinical antibiotics in dogs, the resistance of the common Gram-negative conditional pathogens in the oral and intestinal tract of dogs has become enhanced, and an increasing number of multidrug-resistant oral and intestinal bacteria have emerged [29]. In particular, *Escherichia coli* producing extended-spectrum β-lactamases and plasmid-mediated colistin-resistant bacteria have attracted extensive attention from many research fields, such as bovine mastitic milk, chicken meat, chicken gut microbiota wild birds [30,31,32,33]. Investigating the resistance of colistin-resistant Gram-negative dominant bacteria in the oral and fecal samples of dogs has become one of the important tasks in pet clinical practice and had significant implications for public health in dogs and humans.

By analyzing the colistin-resistance of the oral and fecal bacteria from dog origins in this study, the colistin-resistant *Escherichia coli* strains are defined as the most critical nowadays. Nine antibiotics were frequently used in the treatment of dogs in the animal hospital, including ampicillin, ceftriaxone, trimethoprim/sulfamethoxazole, tetracycline, ciprofloxacin, colistin, imipenem, meropenem and gentamicin, which belonged to the β lactams, cephalosporins, sulfonamides, tetracyclines, quinolones, peptides, and carbapenems [34]. The results of antibiotic susceptibility tests showed a similar drug resistance trend of these *Escherichia coli* from between oral and fecal samples of dog origins, and the *Escherichia coli* strains isolated from dogs with the treatment of antibiotics within the past two months had a higher resistance to nine common antibiotics compared to those from dogs without antibiotics. It suggested that even the use of low-level antibiotics can cause changes in gastrointestinal microbiota and lead to antibiotic resistance issues, which corresponded with the previous research [35]. Because the use of antibiotics has a significant impact on the resistance of the host’s normal microbiota, the continuous increase and expansion of the proportion of drug-resistant bacteria in the host will result in unsatisfactory clinical treatment and prevention, further leading to many medical difficulties [36]. In the multidrug resistance testing, the *Escherichia coli* isolated in this study exhibited high resistance to multiple antibiotics which are commonly used in veterinary clinics, and the proportion of *Escherichia coli* strains that were resistant to both two or more antibiotics even reached 69.96% (538/769). Of major concern is a possible transmission of resistant *Escherichia coli* between animals and humans through numerous pathways, such as the food chain, and direct contact with animal excretions [37]. *Escherichia coli* also represents a major reservoir of resistance genes that may be responsible for treatment failures in both human and veterinary medicine [38]. Hence, multidrug resistance in *Escherichia coli* has become a worrying issue that is increasingly observed in veterinary medicine but also in humans worldwide.

## 4. Materials and Methods

### 4.1. Sample Collection

In mainland China, fecal and oral samples were taken from dogs in animal hospitals, residential areas, and shelters from September 2020 to December 2021. Several basic information about dogs were recorded, including the age, breed, gender, health status, living area and the use of antibiotics. Among them, the living areas nearly covered the entire mainland China, including Northern China (Jilin, Heilongjiang, Liaoning and Neimenggu provinces), Eastern China (Shanghai, and Anhui provinces), Western China (Chongqing province), and Central China (Shangdong, Henan, Beijing and Hebei provinces). The procedures of samples were treated as follows: After the defecation of dogs, fresh feces were immediately obtained into a sterile tube containing 30% glycerol. Alternatively, the fecal collection was performed with veterinarian intervention, which was collected rectally using a sterile cotton swab. The oral swabs mainly scraped the lateral gingival mucosa of dogs. All collected samples were rapidly stored at −80 °C until processing.

### 4.2. Preparation of Conditioned Medium

To isolate the colistin-resistant bacteria, three microbial mediums were prepared in this study, including brain heart infusion (BHI) broth, Salmonella-Shigella (SS) agar plates, and MacConkey agar plates. For the preparation of BHI medium, a total of 38.5 g BHI broth was added to 1000 mL distilled water and performed 121 °C high-pressure sterilization for 15 min. About the SS agar plates, a total of 63.53 g SS agar powder was dissolved in 1000 mL distilled water, with a 110 °C high-pressure sterilization for 20 min and the colistin when the temperature dropped to around 50 °C to pour into the 90 mm disposable plastic culture dish according to the standard of 15 mL/culture dish. To prepare the MacConkey agar plates, 50.0 g MacConkey agar powders were dissolved in 1000 mL distilled water, and were carried on under the same steps after a 121 °C high-pressure sterilization for 15 min. All microbial mediums were stored in a refrigerator at 4 °C to use within a week. 

### 4.3. Screening for Colistin-Resistant Bacteria

For bacterial enrichment, all samples were respectively incubated in BHI broth for 12–16 h at a 37 °C incubator. Subsequently, the volume of 150 μL enrichment samples was, respectively, cultured on the SS and MacConkey agar plate containing colistin (4 μg/mL) for 24 h. Growing bacteria in two agar plates were selected according to their size, shape, and color. As already known, different bacteria have various characteristics in a colony, such as the peach red colonies in *Escherichia coli*, the colorless colonies in Shigella, the black central colonies in Salmonella present, and inhibition in Staphylococcus aureus. A single colony that had different forms was picked with a sterilized wire loop and re-streaked on corresponding agar plates for purification. The process was repeated until the purified single colony was obtained. 

### 4.4. Bacterial Identification

The different colonies representative of each morphological category were picked up from the agar plates and cultured in BHI broth medium for 24 h, separately. To identify the colistin-resistant bacteria, the genomic DNA was extracted from enrichment colonies using DNA Miniprep Kit (Omega, Norcross, GA, USA) according to the manufacturer’s instructions, and PCR amplification was then performed based on a pair of universal primers (27F: 5′- AGAGTTTGATCCTGGCTCAG-3′; 1429R: 5′-GGTTACCTTGTTACGACTT-3′), with a length of about 1500 bp. PCR was conducted in a 25-μL volume mixture which consisted of 12.5 μL 2 × Rapid Taq Master Mix (Vazyme, Biotech Co., Ltd., Nanjing, China), 1 μL sample DNA, 8 μL ddH_2_O, and 0.5 μL each of the primers (25 μM). The reaction was a pre-denaturation at 95 °C for 5 min, 35 cycles of denaturation at 95 °C for 15 s, annealing at 60 °C for 15 s and extension at 72 °C for 30 s and a final elongation step at 72 °C for 10 min. DNA from the sterile BHI broth medium was used as a negative control. PCR products were used to electrophoresis on a 1% agarose gel containing ethidium bromide and visualized under an ultraviolet light transilluminator. The positive PCR products were sent to Comate Bioscience Co., Ltd. in Changchun, Jilin Provence, China, for sequencing in triplicate, and the obtained sequences were aligned to the GenBank database using NCBI BLAST.

### 4.5. Analysis of Prevalence in Colistin-Resistant Escherichia coli from Dog Origin

According to statistical analysis, the isolated rate of colistin-resistant *Escherichia coli* was relatively higher than that of other bacteria of dog origin. To further analyze the resistance of *Escherichia coli* from dog origin to colistin in different factors, the sampling origin, living area and the use of antibiotics in dogs were recorded. The samples mainly were collected from feces and oral swabs of dogs. The dogs in the study were from various regions: Northern China, Southern China, Northern China and Southern China. In addition, it was necessary to investigate whether the dogs had been using antibiotics within two months. 

### 4.6. Antibiotic Susceptibility Tests (AST)

According to the recommended procedure of the Clinical and Laboratory Standards Institute (CLSI), the broth microdilution method was performed to explore antibiotic resistance. Nine antimicrobial agents were used to detect the resistance of the strains isolated in this study, including ampicillin, ceftriaxone, trimethoprim/sulfamethoxazole, tetracycline, ciprofloxacin, colistin, imipenem, meropenem, and gentamicin. The antimicrobial agents evaluated by the microdilution and the dilution ranges tested were listed in Table 6. The panel included only one dilution series for each antimicrobial agent incorporated into the panel. The positive control wells contained no antimicrobial agent, and the negative control remained uninoculated. In susceptibility testing of bacterial strains, the *Escherichia coli* standard strain NCC25922 and *Pseudomonas aeruginosa* standard strain BCC27853 were used as reference strains in this experiment.

### 4.7. Statistical Analysis

The accumulated data, including identification and prevalence, were represented in the respective animal population as relative frequency (percentage). Data were analyzed using Prism 7.0 (Graph Pad Inc., La Jolla, CA, USA), and differences were considered statistically significant when the value of *p* was less than 0.05, with a very significant difference when the value of *p* was less than 0.01.

## 5. Conclusions

Taken together, we first analyzed the current colistin resistance of oral and fecal microbiota from dog origins in mainland China and determined that the most common colistin-resistant bacteria *Escherichia coli* could produce resistance to multiple antibiotics. This study guided guidance on the use of antibiotics in clinical practice and increased the attention to antibiotic resistance in veterinary clinical treatment for animals in the future.

## Figures and Tables

**Table 1:** Information on antibiotic-resistant strains from dog origins in this study.

Sample	Colistin-Resistant Bacteria						Number of Strains	Total	Ratio
	Phylum	Class	Order	Family	Genus	Species			
Feces	Proteobacteria	γ-proteobacteria	Enterobacteriales	*Enterobacteriaceae*	*Enterobacter*	*Enterobacter cloacae*	145	1456	9.96%
					*Escherichia*	*Escherichia coli*	593		40.73%
					*Klebsiella*	*Klebsiella pneumoniae*	256		17.58%
					*Salmonella*	*Salmonella* sp.	63		4.33%
					*Proteus*	*Proteus mirabilis*	399		27.4%
Oral swabs	Proteobacteria	γ-proteobacteria	Enterobacteriales	*Enterobacteriaceae*	*Klebsiella*	*Klebsiella pneumoniae*	86	803	10.71%
					*Escherichia*	*Escherichia coli*	176		21.96%
					*Proteus*	*Proteus mirabilis*	541		67.37%

**Table 2:** Information on colistin-resistant *Escherichia coli* from dog origins in this study.

		The Number of Strains	Ratio	Total
Sampling origin				
	Feces	593	77.11%	769
	Oral swabs	176	22.89%	
Antibiotic usage				
	Yes	102	13.26%	769
	No	667	86.74%	
Regions				
Northern China		334	43.43%	769
	Jilin	298		
	Heilongjiang	20		
	Liaoning	9		
	Neimenggu	7		
Eastern China		101	13.13%	
	Shanghai	53		
	Anhui	48		
Central China		41	22.37%	
	Henan	4		
	Beijing	35		
	Hebei	2		
Western China		162	21.07%	
	Chongqing	162		

**Table 3:** Resistance of *Escherichia coli* from dog origins to different antibiotics.

Antibiotic Classification	Antimicrobial Agent	Abbreviations	Antibiotic Resistance Rate % (Number of Antibiotic-Resistant Strains)		
			All Bacterial Strains (n = 769)	Bacteria from Feces (n = 593)	Bacteria from Oral Swabs (n = 176)
β-lactams	Ampicillin	AMP	57.22% (440/769)	58.35% (346/593)	53.40% (94/176)
Cephalosporins	Ceftriaxone	ZOX	60.6% (466/769)	64.25% (381/593)	48.30% (85/176)
Sulfonamides	Trimethoprim/sulfamethoxazole	SXT	53.06% (408/769)	58.52% (347/593)	34.66% (61/176)
Tetracyclines	Tetracycline	TET	39.27% (302/769)	44.35% (263/593)	22.16% (39/176)
Quinolones	Ciprofloxacin	CIP	34.98% (269/769)	41.82% (248/593)	11.93% (21/176)
Peptides	Myxin	CST	26.92% (207/769)	29.51% (175/593)	18.18% (32/176)
Carbapenem	Imipenem	IMI	15.86% (122/769)	19.06% (113/593)	5.11% (9/176)
Carbapenem	Meropenem	MEM	11.31% (87/769)	13.66% (81/593)	3.41% (6/176)
Aminoglycosides	Gentamicin	CEN	28.35% (218/769)	31.20% (185/593)	18.75% (33/176)

**Table 4:** Resistant rate of *Escherichia coli* from dog origins to different antibiotics.

Items	Antimicrobial Agents (Abbreviations)								
	AMP	ZOX	SXT	TET	CIP	CST	IMI	MEM	GEN
Antibiotic usage									
Yes	67.50	63.29	84.69	58.00	43.06	35.00	32.09	29.47	39.95
No	42.32	51.70	59.02	31.01	35.40	28.40	14.10	12.03	24.80
Regions									
Northern China	50.20	53.05	60.23	30.90	39.56	20.44	15.73	20.44	18.93
Eastern China	58.71	67.02	61.34	40.98	40.72	25.68	38.04	18.72	24.55
Central China	52.04	57.19	60.20	34.23	37.06	27.23	10.72	16.99	21.50
Western China	61.23	65.40	56.07	39.87	41.22	31.90	37.65	21.34	20.89

**Table 5:** *Escherichia coli* resistance spectrum and statistics on these strains.

Number of Drug Resistances	Drug Resistance	Number of Drug-Resistant Bacteria	Ratio (%)	Total
0	—	46	5.98%	5.98% (46/769)
1	AMP	51	6.63%	24.06% (185/769)
1	ZOX	37	4.81%	
1	SXT	28	3.64%	
1	TET	13	1.69%	
1	CIP	19	2.47%	
1	CST	11	1.43%	
1	IMI	7	0.91%	
1	MEM	5	0.65%	
1	GEM	14	1.82%	
2	AMP-ZOX	52	6.76%	28.09% (216/769)
2	AMP-SXT	49	6.37%	
2	ZOX-SXT	33	4.29%	
2	ZOX-CIP	27	3.51%	
2	CST-MEM	13	1.69%	
2	TET-GEM	11	1.43%	
2	CIP-GEM	7	0.91%	
2	SXT-TET	24	3.12%	
3	AMP-ZOX-SXT	36	4.68%	12.36% (96/769)
3	AMP-ZOX-TET	12	1.56%	
3	ZOX-SXT-CST	27	3.51%	
3	SXT-CIP-CST	6	0.78%	
3	AMP-IMI-GEM	7	0.91%	
3	CST-IMI-MEM	2	0.26%	
3	SXT-IMI-GEM	5	0.65%	
4	AMP-ZOX-SXT-CIP	34	4.42%	10.14% (78/769)
4	AMP-ZOX-SXT-CST	15	1.95%	
4	AMP-TET-CIP-GEM	6	0.78%	
4	ZOX-SXT-CST-GEM	11	1.43%	
4	ZOX-SXT-CST-MEM	4	0.52%	
4	TET-CIP-IMI-GEM	8	1.04%	
5	AMP-ZOX-SXT-TET-CIP	29	3.77%	7.41% (29/769)
5	AMP-ZOX-SXT-CIP-GEM	18	2.34%	
5	AMP-ZOX-SXT-CST-MEM	10	1.30%	
6	AMP-ZOX-SXT-TET-CIP-IMI	23	2.99%	4.16% (32/769)
6	AMP-ZOX-SXT-CST-MEM-IMI	9	1.17%	
7	AMP-ZOX-SXT-TET-CIP-MEM-IMI	16	2.08%	3.64% (28/769)
7	AMP-ZOX-SXT-TET-CST-MEM-IMI	12	1.56%	
8	ZOX-SXT-TET-CIP-CST-MEM-IMI-GEM	19	2.47%	2.47% (19/769)
9	AMP-ZOX-SXT-TET-CIP-CST-MEM-IMI-GEM	13	1.69%	1.69% (13/769)

**Table 6:** Antimicrobial agents included in the test panel, range of antimicrobial agent dilutions, and interpretive criteria for *Escherichia coli* isolates.

Antibiotic Classification	Antimicrobial Agent	Abbreviations	Dilution Range (μg/mL)	MIC (μg/mL) for Category	
				S	R
β-lactams	Ampicillin	AMP	0.25–128	≤8	≥32
Cephalosporins	Ceftriaxone	ZOX	8–0.015	≤1	≥2
Sulfonamides	Trimethoprim/sulfamethoxazole	SXT	16/304–0.125/2.375	≤2/38	≥4/76
Tetracyclines	Tetracycline	TET	0.125–64	≤4	≥16
Quinolones	Ciprofloxacin	CIP	8–0.015	≤1	≥2
Peptides	Myxin	CST	8–0.06	≤1	≥2
Carbapenem	Imipenem	IMI	8–0.015	≤1	≥2
Carbapenem	Meropenem	MEM	0.08–0.15	≤1	≥2
Aminoglycosides	Gentamicin	CEN	0.06–32	≤4	≥16

## Data Availability

The data that support the findings of this study are available from the authors, upon reasonable request.
